# A Catheter-Guided Technique for Second Branchial Cleft Fistula Excision in Infants: A Case Report

**DOI:** 10.70352/scrj.cr.25-0013

**Published:** 2025-04-01

**Authors:** Akio Kawami, Yudai Goto, Yuri Nemoto, Tomohiro Aoyama, Kouji Masumoto

**Affiliations:** Department of Pediatric Surgery, Institute of Medicine, University of Tsukuba, Tsukuba, Ibaraki, Japan

**Keywords:** second branchial cleft fistula, branchial cleft anomaly, peripherally inserted central venous catheter, infant

## Abstract

**INTRODUCTION:**

Second branchial cleft (SBC) fistulas are the most common branchial cleft anomalies and typically present in infancy or early childhood. While complete surgical excision is the standard treatment, surgical challenges in younger children arise because of narrower fistula tracts and inadequate visualization, which increase the risk of complications and recurrence.

**CASE PRESENTATION:**

We report the case of a 6-month-old boy with an SBC fistula who presented with persistent mucoid discharge from a right cervical orifice. Fistulography confirmed the presence of a complete SBC fistula. At 9 months of age, a fistulectomy was performed using a 28-gauge peripherally inserted central venous catheter (PICC) as a guide to identify the entire fistula tract. The catheter facilitated the precise identification of the fistula tract, saline irrigation, dissection under endoscopic guidance, and confirmation of the internal end by creating a knot at the catheter tip. The tract was excised completely without any complications. The patient recovered uneventfully and showed no recurrence at 6 months postoperatively.

**CONCLUSIONS:**

Complete surgical resection remains the gold standard for SBC fistulas. However, anatomical challenges in younger children limit the applicability of various intraoperative tools and techniques. The use of a small-diameter catheter as a guide to identify an entire fistula tract is a valuable approach that overcomes these challenges, enhances surgical precision, and reduces the risk of complications and recurrence.

## Abbreviations

PICC
: peripherally inserted central venous catheter
SBC
: second branchial cleft

## INTRODUCTION

Second branchial cleft (SBC) anomalies are the most common in the 4 types of such anomaly, accounting for 80%–95% of cases.^1)^ An SBC fistula opens onto the skin between the lower and higher thirds of the anterior border of the sternocleidomastoid muscle. The fistula travels beneath the sternocleidomastoid muscle, passes through the carotid bifurcation, and reaches the pharynx close to the tonsils. The diagnosis of an SBC sinus or fistula can usually be made clinically; the average age at diagnosis of branchial cleft anomalies is approximately 3–4 years of age, with one-third of patients having a history of infections. However, the branchial cleft sinus or fistula is usually observed in neonates or at an early age.^2)^

Several techniques have been described for the treatment of SBC fistula, such as inside-out tract stripping^3)^ and trichloroacetic acid chemical cauterization of the fistula tract^4)^; however, complete surgical resection remains the most widely accepted treatment modality. In younger children, the smaller and narrower fistula often leads to inadequate visualization, which can increase the risk of complications.

Herein, we report a case with SBC fistula in which excision performed using a peripherally inserted central venous catheter (PICC) as a guide for identifying the entire fistula tract.

## CASE PRESENTATION

**Fig. 1:**
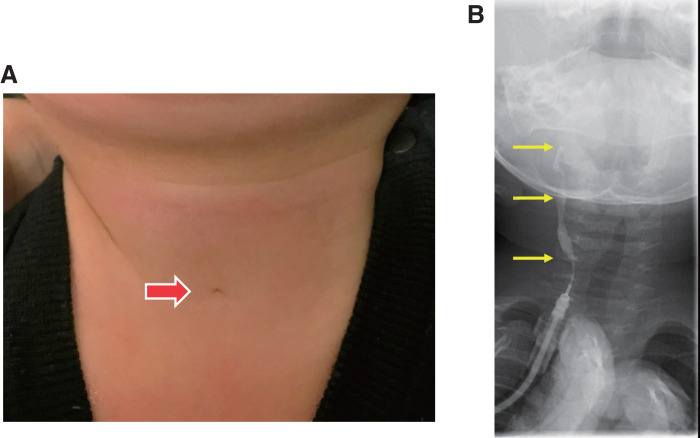
Preoperative findings of this case. (**A**) The patient presented with mucus discharge from a small opening in the skin along the anterior border of the sternocleidomastoid muscle in the right lower neck. No redness or swelling was observed. (**B**) Fistulography performed using a 22-gauge catheter inserted into the fistula showed contrast medium ascending through the fistula to the pharyngeal level and entering the esophagus during swallowing.

A 6-month-old male infant with a persistent mucoid discharge from a right cervical orifice was referred to our hospital. He had no history of infection or cervical abscesses. Physical examination revealed a small opening in the skin at the anterior border of the sternocleidomastoid muscle in the right lower neck (**Fig. 1A**). Fistulography confirmed the diagnosis of a complete SBC fistula (**Fig. 1B**), and a fistulectomy was performed at 9 months of age.

**Fig. 2:**
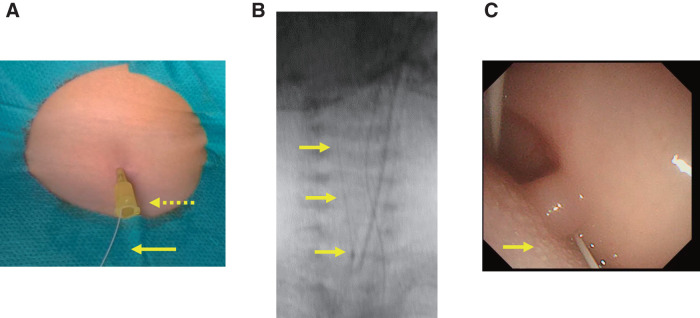
Intraoperative techniques with a peripherally inserted central venous catheter as a guide for identifying the entire fistula tract. (**A**) A peripherally inserted central venous catheter (arrow) was cannulated into the fistula tract, following the insertion of a 24-gauge intravenous (IV) cannula (dotted arrow) through the external opening. (**B**) Under fluoroscopy, the PICC was observed being inserted from the external opening toward the oral side. (**C**) Under endoscopic observation, the catheter was seen emerging from the palatine tonsillar fossa.

**Fig. 3:**
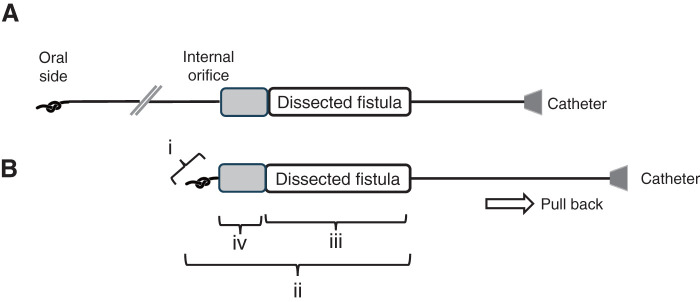
Schematic illustration of knot-tying technique and identification of the internal orifice of the fistula using catheter length. (**A**) The catheter is inserted from the neck, passed through the fistula, and pulled out from the oral cavity. A knot is then created at its end. (**B**) When pulling the catheter back, the knot gets caught at the internal orifice. Gentle traction applied to both the catheter and the fistula creates appropriate tension along the tract, facilitating easier and more precise fistula dissection. The catheter has measurement markings, allowing comparison of the knot position (i), visible catheter length from the neck (ii), and dissected fistula length (iii). This helps estimate the distance (iv) to the internal orifice.

**Fig. 4:**
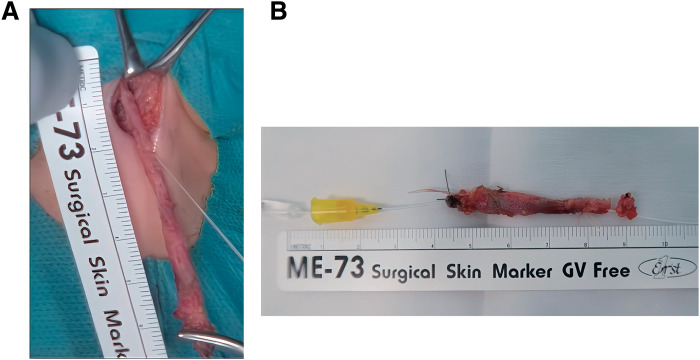
Complete resection of the fistula tract. (**A**) Using the PICC as a guide, the fistulous tract was followed and dissected from surrounding structures to reach the level of its internal end. (**B**) Completely resected specimen. PICC, peripherally inserted central venous catheter

The patient was placed in the supine position with a pillow under the shoulders for neck extension under general anesthesia with endotracheal intubation. Intraoperatively, a 28-guage PICC (Argyle Fukuroi PI catheter; CardinalHealth, Tokyo, Japan) was cannulated through the fistula tract via the external opening following a 24-guage intravenous cannula as an introducer (**Fig. 2A**). Initially, there was resistance when flushing saline through the PICC; however, after continued irrigation, the resistance decreased. Under endoscopic observation, fluid was seen exiting through the internal orifice of the fistula, confirming its patency. Subsequently, the PICC was advanced under oral endoscopic observation and fluoroscopy (**Figs. 2B** and **2C**), which confirmed the external opening was in continuity with the internal opening. The tip of the PICC was pulled out through the mouth, a knot was tied in the distal end, and the catheter was gently pulled back through the external opening of the cervical fistula. A spindle shaped skin incision was made around the opening of the cervical fistula. Using a PICC as a guide, the fistulous tract was dissected from the surrounding structures. The catheter was gently pulled to ensure the knot was secure and the dissection was confirmed to have reached the level of the pharyngeal constrictor muscle under oral endoscopic observation (**Figs. 3A** and **3B**). The entire fistula tract was completely excised (**Figs. 4A** and **4B**), and both internal and cervical incisions were closed without a drain. Histopathological examination revealed that the fistula lumen was lined with stratified squamous and columnar epithelia consistent with an SBC fistula. The postoperative course was uneventful, and the patient was discharged on the fifth postoperative day. No recurrence was observed for 6 months postoperatively.

## DISCUSSION

The standard treatment for SBC fistulas is complete surgical excision of the sinus or fistula tract. The most critical aspects of the procedure are preventing recurrence and avoiding damage to nerves and cervical vessels. Recurrence after operative treatment has been reported to be approximately 3%–6%. However, the recurrence rate may be as high as 22% in patients with a history of multiple infections or after incomplete tract excision due to inaccurate identification of the entire fistula length.^5)^

SBC fistulas are typically observed in young children, with many cases presenting in infancy or early childhood. However, the appropriate timing of excision is controversial. Kajosaari et al. suggested that because the age at operation did not influence the clinical outcome of operative treatment, in patients with no infectious sequelae, the operation may be delayed to an age of approximately 3 years, when anesthesiologic risks and possible harms are best avoided.^2)^ However, Schroeder et al. advocated surgical treatment of branchial anomalies at approximately 1 year of age.^6)^ This view of operative timing is supported by the increase in recurrence if infections precede the operative treatment.

Several assistive methods have been used intraoperatively to assess the entire extent of a second branchial cleft tract. Although methylene blue tract injection is the most universally known method, undue spillage of the dye in adjacent tissues due to excessive injection pressure limits accuracy and can create difficulties in identification during excision.^5)^ Piccioni et al. proposed the injection of fibrin glue combined with methylene blue dye to create a stable dyed gel that prevents the dye from spreading.^7)^ Some studies have reported the usefulness of endoscopic dissection techniques with or without extraluminal visualization; however, these are not applicable for infants and younger children with narrow lumens, and proficiency in endoscopy is a prerequisite for performing the technique.^5)^

Srinath et al. reported that catheterization of the fistulous tract (ureteric catheter) can prevent from extensive dissection, thus making the procedure relatively simple, less time-consuming, and ensuring complete removal of the fistulous tract.^8)^ This technique can also prevent complications encountered with other surgical options, such as injury to the facial nerve and blood vessels of the neck. In fact, Yilmaz et al. reported the use of a 4F catheter through a guide wire inserted intraoperatively under fluoroscopic guidance to aid SBC fistula excision.^9)^

The use of a 28-gauge PICC, as demonstrated in this case, may offer a practical and effective alternative for fistula dissection in young children. This type of catheter is commonly available in many pediatric medical centers and does not require specialized skills, making it a highly accessible tool. Occasionally, the fistula tract may be blocked by thick secretions or granulation tissue,^10)^ and the PICC is particularly useful in such cases because of its ability to irrigate with saline; its soft, flexible material allows it to adapt easily to the tortuous shape of the fistula, and tying a knot at the tip facilitates recognition of the internal orifice of the fistula. These features enable the precise identification of the fistula tract and safe dissection, even in young children with narrow fistulas. Moreover, the combination of endoscopic and fluoroscopic guidance ensures accurate localization and complete excision of the tract, thereby reducing the risk of incomplete resection and operative complications. The PICC has enough strength to prevent tearing during insertion and removal. However, careful handling during surgery is necessary to avoid any damage to the catheter.

## CONCLUSIONS

The use of a small-diameter catheter as a guide for fistula dissection may be a valuable method in the surgical management of SBC fistulas, particularly in young children. This technique provides a reliable method for overcoming the anatomical challenges associated with narrow fistula tracts in younger patients and may contribute to reducing surgical complications and recurrence rates.

## ACKNOWLEDGMENTS

We would like to thank Editage (www.editage.jp) for the English language editing.

## DECLARATIONS

### Funding

No significant financial support or funding was received for this study that could have influenced its outcome.

### Authors’ contributions

AK, YG, and KM conceived the idea for this report.

AK, YG, YN, and TA contributed to the interpretation of intra- and perioperative data.

KM supervised the conduct of this study.

AK and YG significantly contributed to drafting the manuscript.

All authors critically reviewed and revised the manuscript and approved the final version for submission.

### Availability of data and materials

Data sharing is not applicable to this article as no datasets were generated or analyzed during the current study.

### Ethics approval and consent to participate

The need for ethical approval was waived by the Institutional Review Board because this was a case report. Informed consent was obtained from the parents of the patient in this study and recorded in the medical chart at our institution.

### Consent for publication

Informed consent for publication was obtained from the parents of the patient and recorded in the medical chart at our institution.

### Competing interests

The authors declare that they have no competing interests.
